# High-Volume LVA—New Surgical Technique for Treatment of Lymphoceles in the Groin

**DOI:** 10.3390/clinpract16040071

**Published:** 2026-03-31

**Authors:** Daniel Schiltz, Mahsa Bagheri, Stephan Schreml, Philipp Lamby, Adrian Vater, Lukas Prantl, Uwe von Fritschen

**Affiliations:** 1 Department of Plastic and Aesthetic Surgery, Hand Surgery, Helios Hospital Emil von Behring, 14165 Berlin, Germany; 2 Department of Plastic, Hand- and Reconstructive Surgery, University Hospital Regensburg, 93053 Regensburg, Germany; 3 Clinic for Plastic and Hand Surgery, Burn Care Center, Cologne Merheim Medical Center (CMMC), University of Witten/Herdecke, 51109 Cologne, Germany; 4 Department of Dermatology, University Hospital Regensburg, 93053 Regensburg, Germany; 5 Department of Plastic, Aesthetic, Hand- and Reconstructive Surgery, Klinikum Passau, Klinikum des Universitären Medizincampus Niederbayern, Innstr. 76, 94032 Passau, Germany

**Keywords:** lymphocele, lymphovenous anastomosis, lymphatic surgery, microsurgery, high-volume LVA

## Abstract

**Background:** Groin lymphoceles are common postoperative complications after vascular interventions that can be difficult to treat, especially in recurrent or complex cases. While lymphovenous anastomosis (LVA) is a potential, minimally invasive option, its limited flow capacity may not provide sufficient drainage in large lymphoceles. We present a novel high-volume LVA technique that uses larger veins to directly drain the lymphocele cavity. **Methods:** Five patients with six groin lymphoceles, all previously treated unsuccessfully with conventional methods (mean 3.3 surgeries), underwent high-volume LVA (HV-LVA). The technique involved direct anastomosis of a large regional vein to the lymphocele cavity. Lymphatic inflow points were identified with Patent Blue or ICG when possible. Outcomes were assessed over 6–14 months. **Results:** In total, seven HV-LVAs were performed. Three lymphoceles (50%) were successfully treated with a single operation; three required revisions due to venous occlusion. All patients were successfully treated without recurrence. The average number of surgeries per patient was 2.2. **Conclusions:** High-volume LVA may be an effective option for therapy-resistant inguinal lymphoceles, providing greater drainage capacity than standard techniques. Further studies are needed to confirm its long-term efficacy and safety.

## 1. Introduction

The occurrence of lymphoceles is a frequent complication after surgical interventions and vascular catheterization procedures in the inguinal region with a reported incidence of 2–15% [1,2]. In general, lymphoceles are asymptomatic and of limited size [3]. However, a secondary infection can be fatal. Therapy of lymphoceles remains challenging, with a high recurrence rate despite various therapeutic approaches. A relatively simple treatment is percutaneous drainage, often chosen as the first step of treatment with a relatively high success rate of 84%. Although minimally invasive, the treatment duration is often prolonged, averaging 18.2 days (range: 1–93 days) [4]. If this approach proves unsuccessful, percutaneous catheter drainage with sclerotherapy may be considered as an adjunctive or alternative treatment. This procedure, which utilizes various sclerosing agents such as ethanol, povidone-iodine, tetracycline, doxycycline, bleomycin, talc and fibrin glue, has been described in detail [5,6,7]. It has demonstrated improved success rates in the treatment of pelvic lymphoceles [8] and lymphoceles of the groin [9].

For small lymphoceles, chemical sclerotherapy achieves a success rate of 77–90% and a low recurrence rate of 3–7% [8] for groin lymphoceles. However, its efficiency tends to decrease in larger lesions, even after multiple treatment sessions [10].

Intranodal embolization is a further non-surgical intervention reporting promising results in the treatment of groin lymphocele [9,11]. However, the available data is limited, and follow-up periods are often short. Furthermore, all occlusive procedures carry the risk of inducing secondary lymphedema.

Traditionally, surgical intervention is reserved for cases in which sclerotherapy proves ineffective due to its greater invasiveness compared to other available treatments. With increasing experience in the field of less invasive microsurgical treatment options and the growing success of lymphatic surgery, new treatment options have become available [12,13,14]. Lymphovenous anstomoses (LVAs) of the afferent lymphatic vessel can reduce lymphatic flow into the lymphocele or through the lymphatic fistula, thereby promoting resolution and healing [12,15,16,17,18,19]. However, identifying the specific lymphatic vessel draining into the lymphocele is often challenging and its intraoperative location can be difficult. Recently, intraoperative techniques to facilitate the identification of causative lymphatic vessels have been described [20]. Even if LVA is considered a safe procedure, failure of the anastomosis occurs in 0.14% to 6.16% and infection is the most common complication (0.27% to 2.52%) [21].

In our opinion, the drainage achieved through super-microsurgical LVAs is often insufficient to fully resolve the lymphocele, and secondary edema may persist. Given the paucity of available data, we believe that creating a drainage route with a larger caliber than that of conventional LVAs is necessary to ensure the permanent and effective resolution of lymphatic accumulation. For this reason, we have developed a surgical technique with high-volume lymphatic drainage consisting of a large LVA connected directly to the lymphocele and we successfully applied it to five patients. To the best of our knowledge, a high-volume anastomosis has not yet been described in the literature.

## 2. Materials and Methods

The aim was to develop a technique that maximizes drainage, thereby reducing the risk of insufficient treatment, recurrence and secondary lymphoedema. The concept of lymphovenous anastomosis has already been well researched and is considered a safe procedure. Therefore, redirecting lymphatic flow into a vein is considered a sensible strategy, even in the case of a lymphocele. There are usually many larger veins in the inguinal region, e.g., crosse veins of the saphenous vein, the accessory saphenous vein or veins that open into the femoral vein star. To establish a wide communication between the lymphatic and the venous systems, our approach involves directly connecting one of these major veins to the lymphocele. We consider reducing the lymphocele volume before performing the vascular anastomosis to be a crucial first step. Accordingly, our concept is based on a multistage approach.

### 2.1. Technical Approach

**Figure 1:**
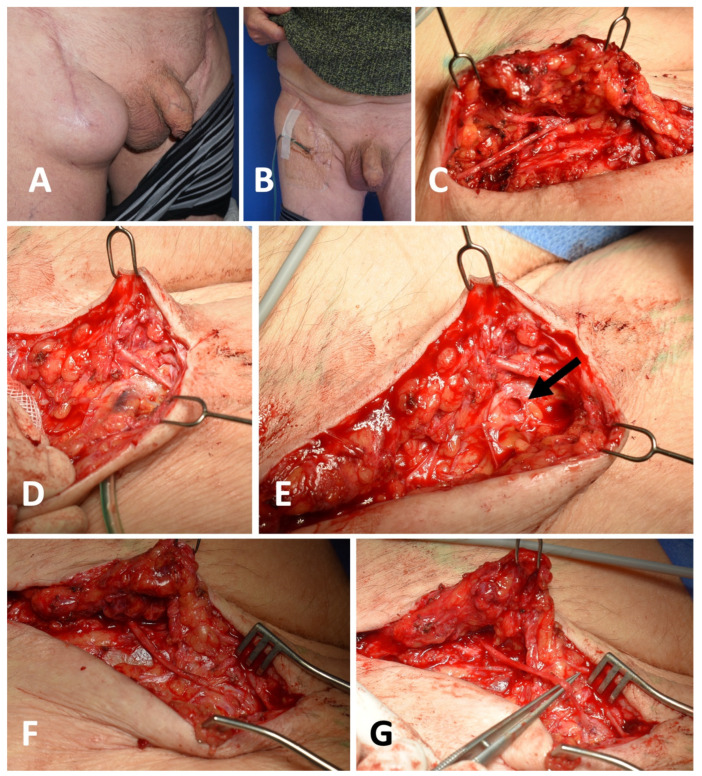
(**A**): Lymphocele in the right groin. (**B**): 14 days after drain insertion in local anesthesia. (**C**): Dissection of the superficial circumflex ileum vein. (**D**): Redon drain entering the lymphocele. (**E**): Lymphatic opening (arrow) in the lymphocele after drain removal. (**F**): Dissected SCIV without retrograde blood flow and positioning for anastomosis. (**G**): High-volume lymphovenous anastomosis.

As a first step, percutaneous drainage of the lymphocele was performed under local anesthesia and sterile conditions using a redon drain (Figure 1B). The drain was left in place for two weeks to promote shrinkage of the lymphocele and to establish a conditioned drainage opening for the subsequent HV-LVA.

**Figure 2:**
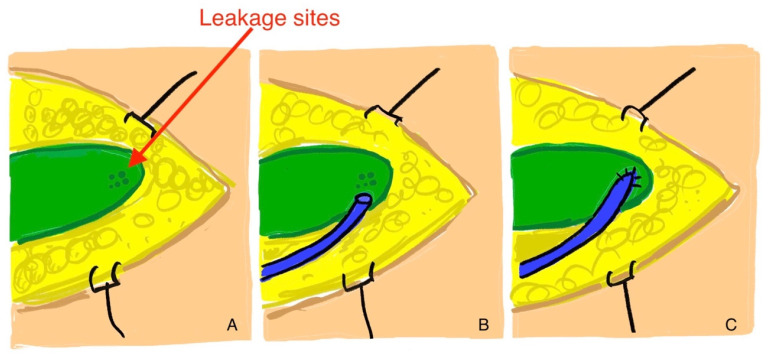
(**A**): Incised lymphocele with visible leakage sites (confluence site). (**B**): Transposition of the vein for the high-volume lymphovenous anastomosis. (**C**): Sutured vein on the leakage site.

The next step was surgical exploration with the aim of creating a lymphovenous anastomosis, if feasible. The procedure was performed two weeks after the drain placement. Patent Blue and ICG (Verdye, Diagnostic Green, Germany) were injected intradermally (interdigital space 1/2 and 3/4 and at the level of the knee joint medially) prior to surgery. During the operation, the lymphocele was opened and inspected. If a single leakage site could be identified in the lymphocele, HV-LVA was applied directly to this site (Figure 2). For this purpose, a suitable vein was anastomosed directly to the leakage site, and the lymphocele was resected as extensively as possible. Sutures were made using a monofilament suture (e.g., Ethilon 6-0 or 8-0, Ethicon, Johnson & Johnson, Cincinnati, OH, USA) (Figure 2C). It is crucial to ensure the absence of retrograde blood flow in the vein. This must be tested prior to suturing to prevent failure of lymphatic drainage. For this reason, preoperative sonographic exclusion of venous insufficiency was performed in each case.

If multiple leakage sites were identified after opening the lymphocele, or if no specific leakage site could be detected, the vein was not connected to the whole lymphocele through its wall. Therefore, the remaining size of the lymphocele must be as small as possible as the larger the remaining volume, the more fibrin deposition and clotting we found due to turbulence or insufficient lymph flow in the lymphocele. If the shrinking effect due to the previously placed redon drain was insufficient, the lymphocele was then resected as much as possible in terms of size. The base of the lymphocele was left intact, as it typically represents the site of lymphatic leakage, and another redon drain was placed into the reduced cavity.

**Figure 3:**
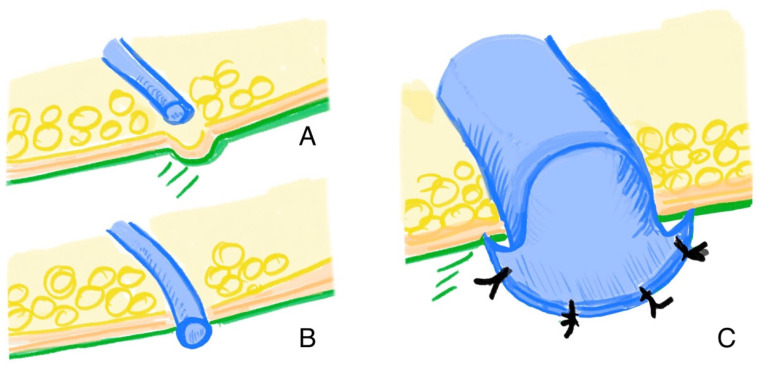
(**A**): Vein placed next to preconditioned opening. (**B**): Inserted vein in the opening of the previously placed drainage. (**C**): Inverted vein in the opening of the previously placed drainage.

**Figure 4:**
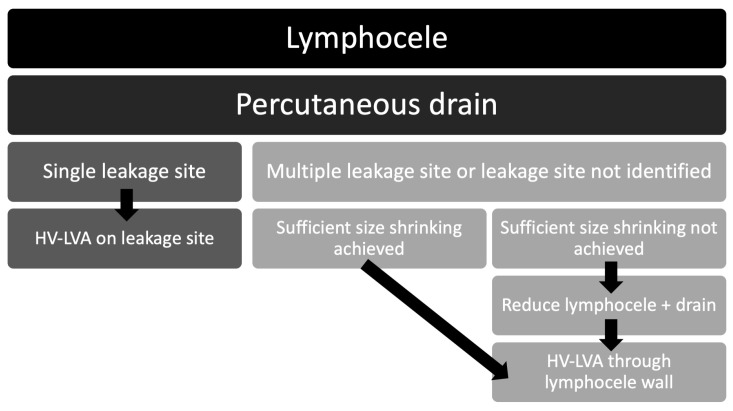
Therapy algorithm for HV-LVA.

In this case, further surgical treatment was necessary. After about a week, the next step was taken once the reduced lymphocele had been conditioned and it had been ensured that there were no further leak sites outside the reduced lymphocele. The drain was removed and a suitable vein was inserted into the lymphocele through the opening of the drain. To ensure that the connection site remained open and to cover the thrombogenic opening edges, the vein was not sutured on the outside of the lymphocele wall, but was placed through the opening and sutured from the inside of the lymphocele wall (Figure 3). Figure 4 shows the therapeutic approach depending on intraoperative findings.

### 2.2. Assessment of Technical and Clinical Success

Recurrent lymphoceles usually occur within a few days to weeks after failed treatment. Therefore, the therapy was considered successful if there was no recurrence 6 months after surgery. To check the technical integrity of the HV-LVA, an ICG scan or CT scan should be performed 6 months after surgery. However, all patients refused postoperative ICG imaging (reasons: off-label use and pain). Only one patient underwent a CT scan.

### 2.3. Study Population

In order to test the new surgical technique, only five patients were initially included in the study. Inclusion criteria were the presence of recurrent lymphocele with clear pathogenesis, the consent of the patients, and the exclusion of venous insufficiency. Exclusion criteria were venous insufficiency, acute infection, and patient refusal.

These five patients had in total 6 lymphoceles in the groin and received 7 high-volume LVAs. One patient had two lymphoceles, one in each groin, following a severe complication of a vascular procedure. All patients had undergone multiple prior surgical interventions, including lymphocele resection and negative pressure wound therapy, before presenting in our department. One patient had a lymphocele with a lymphocutaneous fistula. None of the patients suffered from an acute infection. All patients were treated at the department of Plastic and Aesthetic Surgery, Hand Surgery, Helios Hospital Emil von Behring in Berlin, Germany. Informed consent was obtained from all patients. Descriptive data of the patient collective are shown in Table 1.

## 3. Results

**Figure 5:**
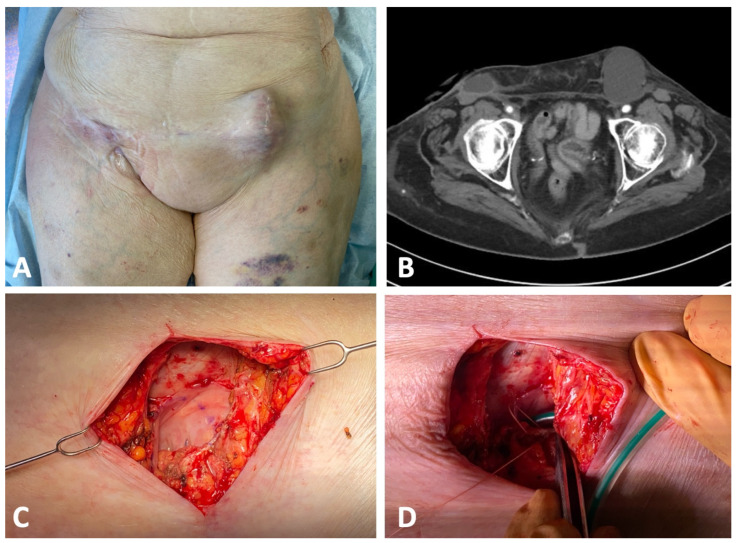
Patient suffering from one lymphocele in each groin. (**A**): Open lymphocele in the right groin; closed lymphocele in the left groin. (**B**): CT scan of the patient. (**C**): Reduced left lymphocele after partial resection. (**D**): Reduced lymphocele with inlying drain.

**Figure 6:**
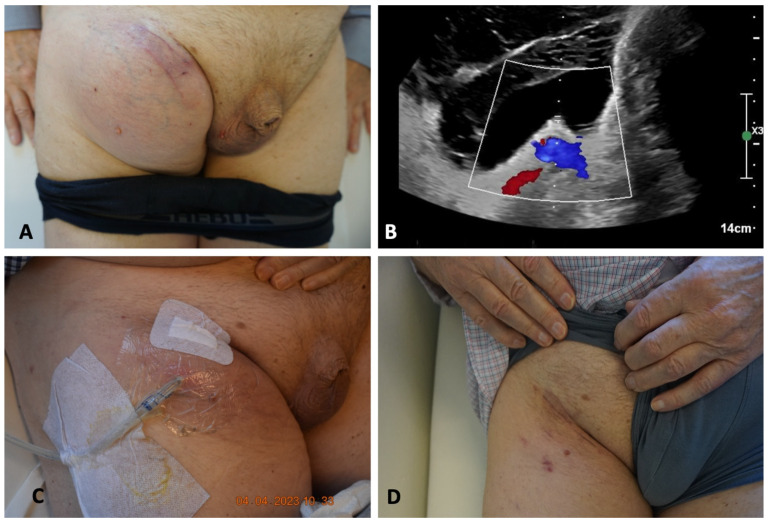
(**A**): Before surgery. (**B**): Ultrasound imaging of the lymphocele above the femoral vessels before HV-LVA. (**C**): After insertion of a drain in local anesthesia. (**D**): 3 months after HV-LVA (SCIV).

The patients needed an average of 2.2 surgeries until successful treatment. Three patients (three lymphoceles) could successfully be treated with only one surgery. In three cases (50% of the lymphoceles), a revision was necessary. In all cases, the reason for the failure of the procedure was the occlusion of the vein. In one of the first cases, the lymphocele was not reduced prior to the HV-LVA. After the reduction of the lymphocele, using the same vein, the procedure was successful. In the other cases, the venous occlusion was presumed to result from the anastomotic technique. Initially, the vein was connected directly to the opening of the lymphocele, on the outer surface of its wall. The exposed cut edges of the lymphocele wall were considered the likely trigger of the thrombotic event. After revising the anastomosis and repositioning the vein through the opening (as shown in Figure 3), thereby ensuring that the incised edges of the lymphocele wall were no longer exposed, the procedure was successful. The patient with two lymphoceles underwent a total of six surgeries. The lymphoceles were large in size (left groin: 14 × 8 × 7 cm, right groin: 3 × 2 × 5 cm) and exhibited multiple leakage points, not all of which could be identified (Figure 5). In total, three HV-LVAs could be performed. The remaining leakage points were closed in further surgeries by surgical clips. During these revision surgeries, the functionality of the HV-LVA could be confirmed by patency test. All patients were successfully treated without recurrence after a follow up period of 9–30 months (mean 15.6 month) (Figure 6). Table 2 summarizes these results.

## 4. Discussion

**Figure 7:**
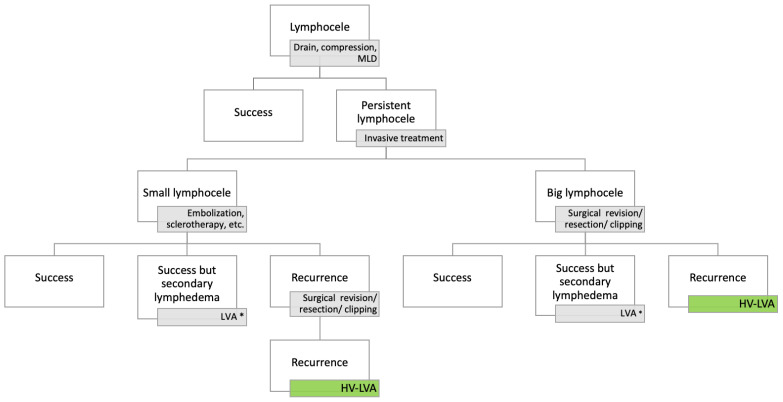
Recommended therapy algorithm. There is no recommendation in the literature regarding the size at which surgical treatment should be sought. * In case of secondary lymphedema, spontaneous resolution should first be allowed initially. LVA should be considered 6 months after conservative therapy-refractory edema. MLD = manual lymphatic drainage; LVA = lymphovenous anastomosis; HV-LVA = high-volume lymphovenous anastomosis.

The novel high-volume lymphovenous anastomosis (HV-LVA) technique presented in this case series offers a promising new approach for the treatment of therapy-resistant groin lymphoceles. In patients with multiple previous surgeries, scar tissue formation, or large lymphoceles, conventional treatments such as drainage, sclerotherapy, or standard super-microsurgical LVA often yield unsatisfactory outcomes. Our findings suggest that connecting a large-caliber vein directly to the lymphocele allows for effective and sustained lymphatic drainage, even in complex and recurrent cases. We recommend surgical therapy as soon as the lymphocele has a size that is functionally disruptive or symptomatic. We therefore recommend the therapy algorithm presented in Figure 7.

All patients in our cohort achieved complete resolution without recurrence after an average of 2.2 surgical procedures. However, approximately half of the cases required revision surgery, mostly due to early venous occlusion. Our analysis suggests that the surgical design of the anastomosis plays a critical role in long-term success. Specifically, routing the vein through a preconditioned opening (Redon drain placed prior to surgery) in the lymphocele wall and avoiding direct exposure of incision edges appears to significantly reduce the risk of occlusion. Later procedures that implemented these modifications demonstrated improved outcomes.

Compared to traditional treatments, HV-LVA offers several potential advantages. It is less invasive than complete surgical excision that carries the risk of creating further lymphatic leakage. Further, it allows for higher lymphatic flow (vein diameter > 2.5 mm) than standard super-microsurgical LVAs (vein diameter ca. 0.5–1.5 mm) and avoids aggressive tissue resection or chemical irritation, thus preserving surrounding structures and reducing the risk of secondary lymphedema. When leaking lymphatic vessels can be identified, the “packing of the groin” technique [22] might be a surgical alternative, although it is not a physiologic approach and carries the risk of inducing secondary lymphedema.

Compared with conventional super-microsurgical LVA, a high-volume anastomosis may carry an increased risk of venous backflow-induced thrombosis. During the follow-up period for our patients, no thrombosis of the femoral vein occurred. There was also no recurrence of the lymphocele, which suggests that the anastomosis remained open and no thrombosis in the connected vein occurred. This was confirmed radiologically in one patient by means of a CT scan. The reason for this might be the increased pressure in the lymphatic system that exceeds pressure in the venous system while venous valves are sufficient. With increasing edema and lymphatic congestion, the pressure in the lymphatic vessels rises [23]. Sonographic confirmation of sufficient veins and valves should therefore be performed preoperatively.

Nevertheless, current evidence on HV-LVA remains limited. Our case series comprises only five patients and six lymphoceles, which restricts the generalizability of the findings. Moreover, it remains unclear whether this technique is equally effective in smaller or less complex lymphoceles, or in anatomical regions other than the groin. Intraoperative identification of lymphatic inflow points using Patent Blue or ICG appears to enhance the accuracy and efficacy of the anastomosis and warrants further investigation [24]. ICG can be used not only intraoperatively to demonstrate the patency of the anastomosis, but also as a follow-up tool. For example, staging of the edema or measurement of the ICG transit time before and after the operation can detect a possible reduction in the load on the lymphatic system and an improvement in lymphatic drainage. Depending on the device and the tissue depth of the applied HV-LVA, it may even be possible to visualize the HV-LVA directly.

Furthermore, we must admit that three out of five patients required follow-up surgery. This was due to the learning curve and the mistakes made initially. For example, at the beginning, the vein was not passed through the wall of the lymphocele and inverted, so that the endothelium-free lymphocele wall was exposed in the lumen and was seen as the reason for the occlusion. However, we assume that the failure rate will decrease with increasing experience and that we will hopefully be able to successfully treat patients in the future with just one well-planned and well-prepared operation. Nevertheless, it must be acknowledged that all patients were successfully treated using this new technique and were spared further operations, potential infections, or chronic lymph fistulas, which would have posed a significant health risk and considerably reduced their quality of life.

Another limitation is the moderate follow-up period (6–14 months). Although no recurrences were observed during this time, longer-term studies are needed to confirm the durability of treatment success. A prospective, comparative study evaluating HV-LVA against conventional LVA, sclerotherapy, or surgical excision would help better define its role within the treatment algorithm. Patient-reported outcomes such as quality of life and postoperative symptoms were not systematically assessed and should be incorporated into future studies. In addition, future research should aim to establish standardized criteria for patient selection, surgical technique, and postoperative care. Furthermore, the potential of preoperative lymphangiography warrants further investigation to optimize surgical planning and outcomes.

## 5. Conclusions

High-volume LVA may become an important therapeutic option for persistent or recurrent groin lymphoceles, especially in patients with extensive surgical histories or contraindications to other procedures. The technique requires microsurgical expertise and must be tailored to individual anatomy, but early results suggest it offers a durable and physiological solution for lymphatic decompression.

## Figures and Tables

**Table 1:** Descriptive data of the patient collective.

Gender	male: *n* = 4 patients female: *n* = 1 patient
Age (mean)	78 years
Duration of lymphocele before presentation (mean)	8.2 weeks
Number of surgical revisions prior to presentation (mean)	3.3 (range 2–4)
Cause	complication of a vascular procedure in all cases

**Table 2:** Summary of the results of high-volume LVAs on lymphoceles. The smallest lymphocele was 30 cm^3^, whereby this was the open lymphocele. All other lymphoceles were larger than 12 × 9 × 4 cm. All used veins had a diameter larger than 2.5 mm. SCIV = superficial circumflex ileum vein; SIEV = superficial inferior epigastric vein.

	Number	Range
Number of surgeries until success	2.2 (mean)	1–6
Number of early recurrence	2	
Number of recurrence rate after revision	0	
Size of lymphocele	1689 cm^3^ (mean)	30–5780 cm^3^
Veins used for LVA: - SCIV - SIEV - lat. accessory vein of the saphena magna vein - crosse vein	3 2 1 1	
Hospital in stay	13.5 days	2–45

## Data Availability

The raw data supporting the conclusions of this article will be made available by the authors on request.

## Abbreviations

The following abbreviations are used in this manuscript:

LVA	Lymphovenous anastomosis
HV-LVA	High-volume lymphovenous anastomosis
MLD	manual lymphatic drainage
SCIV	superficial circumflex ileum vein
SIEV	superficial inferior epigastric vein
